# Detection of cytokine protein expression in mouse lung homogenates using suspension bead array

**DOI:** 10.1186/1476-9255-3-15

**Published:** 2006-11-29

**Authors:** Eric McDuffie, Leslie Obert, Jonathan Chupka, Robert Sigler

**Affiliations:** 1 Drug Safety Evaluation Department, Esperion, a Division of Pfizer Research and Development, Ann Arbor, Michigan, 48108 USA; 2 Worldwide Safety Sciences Department, Pathology Section, Pfizer Research and Development, Ann Arbor, Michigan, 48105 USA

## Abstract

**Background:**

The objective for this present study was to determine whether or not suspension bead array is a feasible method to detect changes in cytokine protein expression in mouse lung tissue homogenates. Here, we report on suspension bead array as a feasible method for detection of lipopolysaccharide (LPS)-evoked changes in cytokine protein expression in mouse lung tissue homogenates.

**Materials and methods:**

Mice were treated (0.2 ml, intraperitoneal, i.p.) with phosphate buffered saline (PBS) or LPS (0.25 mg/ml) and sacrificed at either 2- or 24-hours post treatment. Formalin-fixed and paraffin-embedded lung sections were evaluated by light microscopy. Flash frozen lung tissues were homogenized for measurement of various cytokine protein expression levels using suspension bead array, antibody array and ELISA. Comparison between groups was performed using a Wilcoxon signed rank test.

**Results:**

Pulmonary perivascular edema and an accumulation of mixed cell infiltrates within blood and lymphatic vessels, as well as in the adjacent interstitium, were present at both 2- and 24-hours following LPS treatment. A minimal increase in the number of alveolar macrophages was also observed in the 24-hour LPS-treated mice only. The suspension bead array assay revealed statistically significant increases in mouse lung tissue homogenate levels of interleukin-6 (IL-6) and granulocyte/macrophage colony-stimulating factor (GM-CSF) proteins and a decrease in IL-2 protein at 24-hours post LPS-treatment only. Similar cytokine protein expression patterns were observed using antibody array. Significantly increased IL-6 protein expression levels were also detected using ELISA, which correlated with the suspension bead array data.

**Conclusion:**

The present study shows that suspension bead array is a feasible method to detect changes in cytokine protein expression in mouse lung tissue homogenates.

## Background

The chemically purified endotoxin, lipopolysaccharide (LPS) has no or only traces amounts of cell wall proteins [1]. Alveolar macrophages have a primary role in mediating the effects of LPS entering airways and lungs. CD-1 mice are relatively sensitive to LPS treatment [2,3]. Previous reports suggested that similar to that seen with systemic inflammatory response in CD-1 mice, pro-inflammatory cytokines, interleukin-6 (IL-6) and macrophage inflammatory protein-2 (MIP-2) protein levels in mouse lung tissue homogenates are increased following LPS treatment as determined by ELISA [2]. Increased neutrophil infiltration is also detectable in lung sections in parallel with increased IL-6 and MIP-2 expression in lung tissue homogenates from LPS-treated mice. Additional studies are needed to further elucidate diagnostic markers indicative of the LPS-evoked lung inflammatory injury response.

While, traditional immunohistochemical staining methods for detection of cytokine protein expression in tissue sections is semi-quantitative, current and emerging quantitative methods and their applications including the use of tissue homogenates have been reviewed [3]. Suspension bead array affords multiplexing of microspheres labeled with fluorescent dyes and contain surface carboxyl groups for covalent attachment analytes (i.e., cytokine proteins) to conform a solid phase sandwich immunoassay for measuring multiple analytes simultaneously. We determined in our laboratory that suspension bead array is a useful tool for measuring mouse plasma levels of cytokine protein expression following LPS treatment [4]. In LPS-treated female CD-1 mice, increased plasma levels of IL-6, interleukin-10 (IL-10), interferonγ (IFNγ) and tumor necrosis factorα (TNFα) were detectable at both 2-hours and 24-hours post treatment; while increased plasma protein levels of interleukin-1β (IL-1β), interleukin-5 (IL-5), interleukin-12 (IL-12) and granulocyte/macrophage colony-stimulating factor (GM-CSF) were detectable at 24-hours post-treatment only. Whether or not suspension bead array can be used to measure mouse lung tissue homogenate levels of cytokine protein expression has not been reported in the literature. Procedures for simultaneous measurement of multiple cytokines in rat serum and brain tissue by suspension bead array have been optimized [5,6]. Methods for antibody array analysis of peripheral and blood cytokine levels in rats after masseter inflammation induced by injection of complete Freund's adjuvant have also been described [7].

The objective for this present study was to determine whether or not suspension bead array is a feasible method to detect changes in cytokine protein expression in mouse lung tissue homogenates.

The feasibility of simultaneous measurement of cytokine levels may lend to the elucidation of potential diagnostic markers of compound-induced inflammatory responses in tissues.

## Materials and methods

### Materials

Six to eight-week-old female CD-1 mice were purchased from Charles River Laboratories (Wilmington, MA). Animals were housed in accordance with the current guidelines for animal welfare (Guide for the Care and Use of Laboratory Animals, 1996, Animal Welfare Act, 1996, as amended in 1970, 1976 and 1985, 9 CFR Parts 1,2,3).

The following materials were used: lipopolysaccharide (LPS, Sigma, St. Louis, MO, USA, L2630); phosphate buffered saline (PBS, Sigma); Tissue Protein Extraction reagent (T-PER, Pierce, Rockford, IL, USA); Complete Mini Protease Inhibitor Cocktail tablets (Roche Diagnostics, Indianapolis, IN, USA); bicinchoninic acid (BCA) kit (Pierce); Mouse Cytokine 10-Plex kit, Biosource International, Camarillo, CA, USA); RayBio Mouse Cytokine Antibody Array kit (Panomics, Redwood City, CA, USA); enhanced chemiluminescence (ECL) kit (Amersham Pharmacia Biotech, Piscataway, NJ, USA); X-OMAT AR film (Amersham Pharmacia Biotech); and mouse IL-6 ELISA kit (R&D Systems, Minneapolis, MN, USA).

### Lipopolysaccharide Administration

Twenty mice were assigned to 4 experimental groups (5 animals/group). Dosing solutions of LPS were prepared in PBS. Animals were dosed with 0.2 ml of 0.25 mg/ml LPS or PBS by single intraperitoneal (i.p.) injection, observed for clinical signs immediately post-treatment and prior to sacrifice. Animals were fasted 2-hours prior to sacrifice.

### Lung Harvest and Preparation of Homogenates

Post sacrifice, the left lung lobes were perfused with 10% neutral-buffered formalin containing phosphatase inhibitor cocktail (200 mM Sodium fluoride and 200 mM Sodium pervanadate), placed in fixative for approximately 24 hours, stained for hematoxylin and eosin, and evaluated by light microscopy. The right lung lobes were snap-frozen (in liquid nitrogen) and stored at -80°C until further analysis. The snap-frozen lungs were thawed, weighed, transferred to different tubes on ice containing 1 ml of T-PER containing Complete Mini Protease Inhibitor Cocktail tablets at a proportion of 1 tablet/10 ml of T-PER stock reagent. The lung tissues were homogenized at 4°C. Lung homogenates were centrifuged at 9,000 × g for 10 minutes at 4°C. Supernatants were transferred to clean microcentrifuge tubes, frozen on dry ice and thawed on ice. Total protein concentrations in the lung tissue homogenates were determined using a BCA kit. Lung tissue homogenates were diluted with 50% assay diluent (provided in the Mouse Cytokine 10-Plex kit) and 50% T-PER reagent to a final protein concentration of 500 μg/ml.

### Luminex Suspension Bead Array

The Mouse Cytokine 10-Plex kit reagents were used at 25°C according to the user manual to evaluate interleukins -1β, -2, -4, 5, -6, -10, -12 [p40], -12 [p40] monomer, and -12 [p70], IFNγ, GM-CSF and TNFα expression patterns. Serial dilutions of the lyophilized standard were prepared in assay diluent: T-PER reagent (1:1) and transferred to appropriate microtiter wells containing diluted antibody-coated bead complexes and incubation buffer. 50 μl of each homogenate sample was transferred to appropriate wells containing diluted antibody-coated bead complexes and incubation buffer. Samples were incubated for 2 hours. After washing with assay wash buffer (200 μl/well), 100 μl diluted biotinylated secondary antibody was added to the appropriate wells and incubated for 1 hour. After washing, 100 μl Streptavidin-phycoerythrin was added to each well and incubated for 30 minutes. After a final wash, the plate was analyzed using the Luminex 100 analyzer (Luminex Corp., Austin, TX). Minimums of 400 events (beads) were collected for each cytokine/sample and median fluorescence intensities were obtained. Cytokine concentrations were calculated based on standard curve data using MasterPlex™ QT Analysis version 2 (MiraiBio, Alameda, CA). The results are expressed as mean ± SE (n = 5). Comparisons between all groups for each respective cytokine were performed by analysis of variances, choosing *p < 0.05 *as significant. For IL-2 and GM-CSF, comparison between groups was performed using a Wilcoxon signed rank test at each respective time point, with significance at *p < 0.05*. For IL-6, a two-tailed t-test was applied at the 95% confidence level (*p < 0.05*) at the 24-hour time point only. Analyses were performed using Graphpad Prism version 4 (Graphpad Software, San Diego, CA).

### Antibody Array

Lung homogenate proteins were quantified using a BCA kit. TranSignal™ qualitative measurement of tissue cytokine expression was performed on lung homogenate samples from the 24-hour control (n = 4) and LPS-treated (n = 4) groups using the RayBio Mouse Cytokine Antibody Array kit according to the user manual. The signals were detected using the ECL kit according to the user manual, exposed on X-OMAT AR film and digitized using the Duoscan HiD scanner (AGFA, Wilmington, MA).

### ELISA

IL-6 levels were evaluated in lung tissue homogenates from the 24-hour control (n = 4) and LPS-treated (n = 4) groups using a mouse IL-6 ELISA kit according to the user manual. Comparison between groups was performed using a Wilcoxon signed rank test using Graphpad Prism version 4.

## Results

### Histopathology

**Figure 1:**
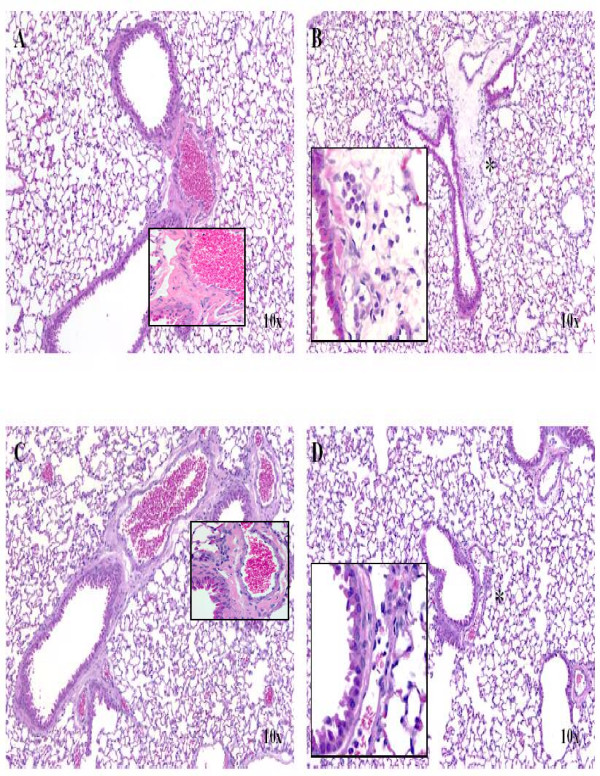
**Representative histology of LPS-induced lung injury in CD-1 mice**. H&E staining of left lung lobe from a female mouse following a single i.p. injection of 0.2 ml of 0.25 mg/ml LPS for 2 hours (B) or 24 hours (D) or PBS (vehicle) for 2 hours (A) or 24 hours (C). No histopathologic findings were observed in the lungs of 2-hour PBS-treated control mice (A). One 24-hour PBS (vehicle)-treated control mouse (C) had a focal incidental mononuclear cell infiltrate present perivascularly within the lung. Perivascular edema and the accumulation of mixed cell infiltrates within blood and lymphatic vessels, as well as in the adjacent interstitium at both 2-hours (B) and 24-hours (D) following LPS treatment. A minimal increase in the number of alveolar macrophages (asterisk) was also observed in the 24-hour LPS-treated mice.

As shown in Figure 1, histopathologic evaluation of the lungs revealed perivascular edema and an accumulation of mixed cell infiltrates within blood and lymphatic vessels, as well as in the adjacent interstitium at both 2-hours (B) and 24-hours (D) following LPS treatment. A minimal increase in the number of alveolar macrophages was also observed in the 24-hour LPS-treated mice. One 24-hour PBS-treated control mouse (C) had a focal incidental mononuclear cell infiltrate present perivascularly within the lung. No histopathologic findings were observed in the lungs of 2-hour PBS-treated control mice (A).

### Suspension Bead Array

**Figure 2:**
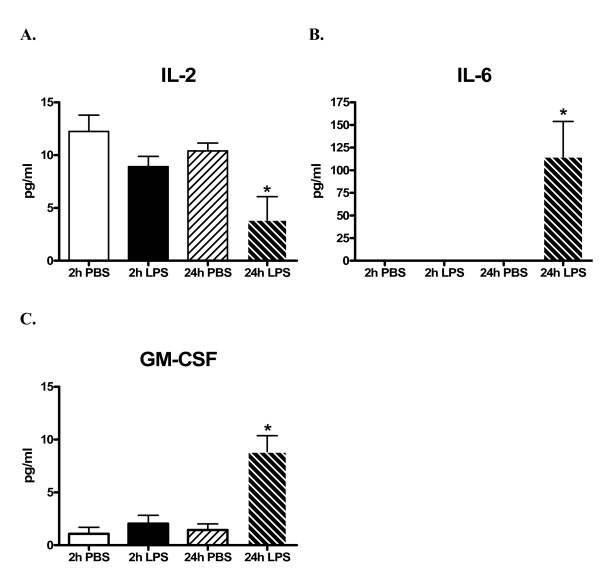
**The alignment of the 22 analytes on the RayBio Mouse Cytokine Antibody Array**. Abbreviations: bFGF: basic fibroblast growth factor; CD: cluster of differentiation; GCSF: granulocye colony stimulating factor; GM-CSF: granulocyte/macrophage-colony stimulating factor; IFN: interferon; IL: interleukin; MCP: monocytes-chemoattractant protein; M-CSF: macrophage-colony stimulating factor; MIP: macrophage inflammatory protein; RANTES: regulated on activation, normal T-cell expressed and secreted; TNF: tumor necrosis factor; VEGF: vascular endothelial cell growth factor. Quantitative levels of cytokine protein concentrations (pg/ml) in lung homogenates from CD-1 mice at both 2- and 24-hours after i.p. administration of LPS or PBS vehicle (control) using suspension bead array. Significant levels of cytokine protein expression increases in lung homogenates from LPS-treated mice were noted for IL-6 (B) and GM-CSF (C) and a significant decrease in IL-2 (A) at 24-hours. Results are represented as the mean (pg/ml) ± SEM. * : P < 0.05 compared to corresponding control. N = 5/treatment group.

As shown in Figure 2, statistically significant cytokine protein expression increases in lung tissue homogenates from LPS-treated mice were noted for IL-6 (574-fold, *p = 0.0425*, B) and GM-CSF (8-fold, *p = 0.0313*, C) and a decrease in IL-2 (1.3-fold, *p = 0.0313*, A) at 24-hours.

### Antibody Array

**Figure 3:**
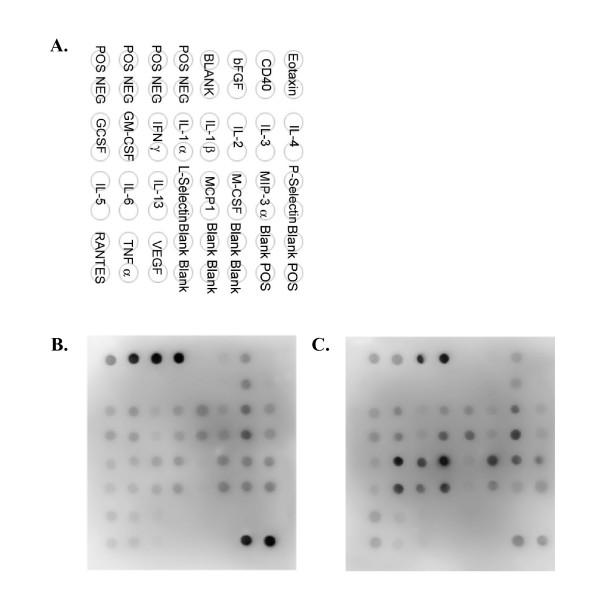
**Representative qualitative levels of cytokine protein expressions in lung homogenates from CD-1 mice at 24-hours after i.p. administration of LPS or PBS vehicle (control) using antibody arrays**. The array key represents the location of each antibody in duplicate on the membrane (A). Increased protein expression levels of IL-6 and IL-13 and L-selectin and decreased IL-2 were detected in lung homogenates from LPS-treated (24-hour) mice (C) but not in lung homogenates from PBS vehicle (control)-treated (24-hour) animals (B). N = 4/treatment group.

Visual interpretation of the TranSignal™ RayBio Mouse Cytokine Antibody Arrays (Figure 3A) suggests increased expression of IL-6, IL-13 and L-selectin and macrophage colony stimulating factor (MCSF) proteins and decreased IL-2 protein in lung tissue homogenates from 24-hour LPS-treated mice (C) compared to PBS-treated control mice (B).

### ELISA

**Figure 4:**
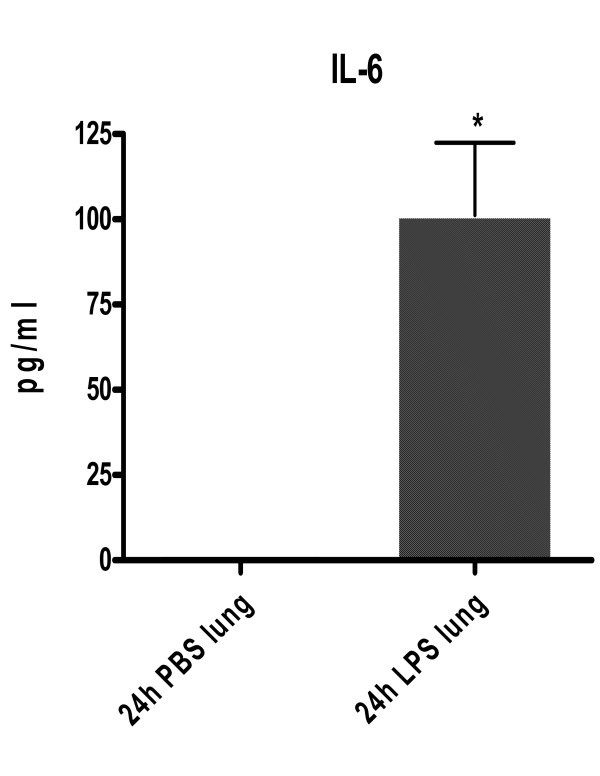
**Quantitative level of IL-6 protein expression in lung homogenates from CD-1 mice at 24-hours after i.p. administration of LPS or PBS vehicle (control) using ELISA**. Results are represented as the mean (pg/ml) ± SEM. * : P < 0.05 compared to corresponding control. N = 4/treatment group.

Similar to the protein expression levels detected using suspension bead array, statistically significant *(p = 0.0313) *increased IL-6 protein expression was detected in lung tissue homogenates from 24-hour LPS-treated mice using ELISA (Figure 4).

## Discussion

CD-1 mice are relatively sensitive to endotoxin and the relative effects of endotoxin (specifically LPS) on lung inflammation in CD-1 mice have been previously described [2]. The purpose of the present study was to investigate the feasibility of using suspension bead array for detection of LPS (i.p. administration of 0.25 mg/ml)-evoked changes in levels of cytokine protein expression (at 2- and 24-hours post treatment) in mouse lung tissue homogenates. Simultaneous detection of changes in levels of lung cytokine proteins could prove useful during the preclinical safety evaluation phase of compound development.

LPS treatment was well tolerated in all animals; and there were no adverse clinical observations in this study. LPS-related histopathology changes were identified in the left lung lobe in female CD-1 mice after treatment with LPS (0.25 mg/ml) for both 2- and 24-hours. The inflammatory changes were characterized by perivascular edema and the accumulation of mixed cell infiltrates within blood and lymphatic vessels, as well as in the adjacent interstitium at both 2-hours and 24-hours following LPS treatment. A minimal increase in the number of alveolar macrophages was also observed in the 24-hour LPS-treated mice. No histopathology findings were observed in the lungs of 2-hour PBS-treated control mice.

In the present study, among the 24-hour LPS-treated animals, increased IL-6 (574-fold) and GM-CSF (8-fold) and decreased IL-2 (1.3-fold) protein concentrations in lung homogenates were detected using suspension bead array. Previously, we reported observations of statistically significant increases in CD-1 mouse plasma IL-6 protein expression at both 2- and 24-hours post LPS treatment and GM-CSF protein expression at 2-hours post LPS treatment [4]. Suspension bead array-detectable changes in lung homogenate levels of IL-2, -6 and GM-CSF protein expression 24-hours following LPS treatment correlated with qualitative protein expression profile data (i.e., as low as 10 pg/ml of IL-2) detected using antibody array. In this study, antibody arrays findings suggested significantly increased expression of IL-6 and -13 and L-selectin proteins and significantly decreased IL-2 protein in lung homogenates from 24-hour LPS-treated mice. Similar to the protein expression levels detected using suspension bead array, significantly increased IL-6 protein expression was detected in lung homogenates from 24-hour LPS-treated mice using ELISA.

LPS exerts adjuvant effects on macrophages, resulting in an inflammatory cascade defined by early production of TNFα followed by subsequent production of IL-1, IL-6 [8] and MIP-2 [2]. The production of TNFα by macrophages and the recruitment of inflammatory cells are regulated by T cells. High dose (1000 μg/ml) LPS exposure causes activation of both T cells and macrophages, while low dose (100 μg/ml) treatment evokes activation of macrophages only [8]. Perivascular edema and accumulation of mixed cell infiltrates in the lung is typical of lung inflammatory responses [2]. In the present study, the LPS dose of 250 μg/ml evoked lung perivascular edema, accumulation of mixed cell infiltrates within blood and lymphatic vessels and in the adjacent interstitium. These changes parallel the significantly increased levels of IL-6 and GM-CSF in lung tissue homogenate from LPS-treated mice versus controls. Only a minimal increase in the number of alveolar macrophages was observed 24-hours post LPS treatment, which also corroborates with the "minimally" elevated levels of macrophage-origin cytokines (IL-6 and GM-CSF) in lung tissue homogenates. Taken together, the enhanced cytokine levels detected in lung tissue homogenates suggest an increased local cytokine production in the lungs by LPS-activated macrophages. Alternatively, histopathology changes observed in the LPS-treated mice may reflect local effects due to the highly elevated circulating cytokines (TNFα, IFNγ, GM-CSF, IL-1β, IL-5, IL-6, IL-10, IL-12), which were reported previously for these animals [4]. Circulating cytokine levels were log-folds higher than those observed for the lung tissue homogenates, which may indicate a tissue dilution effect and/or a tissue sampling phenomenon, since the histopathology changes were multi-focal and not diffusely distributed. Similarly, the decrease in IL-2 expression in lung tissue homogenates following LPS treatment could possibly be due to tissue dilution effect and/or a tissue sampling phenomenon, but requires further investigation.

In summary, we found that a single i.p. administration of LPS at 0.25 mg/ml yielded significantly increased IL-6 and GM-CSF proteins and decreased IL-2 protein expression in lung tissue homogenates using suspension bead array. These findings correlated with cytokine protein expression profiles detected using antibody array and were confirmed using conventional ELISA (IL-6 protein concentrations).

## Conclusion

Overall, the present study shows that suspension bead array is a feasible method for detecting changes in cytokine protein expression in mouse lung tissue homogenates.

## Abbreviations

lipopolysaccharide, LPS; intraperitoneal, i.p.; phosphate buffered saline, PBS; interleukin, IL; macrophage inflammatory protein-2, MIP-2; interferonγ, IFNγ ; tumor necrosis factorα, TNFα ; granulocyte/macrophage colony-stimulating factor, GM-CSF; tissue protein extraction reagent, T-PER; bicinchoninic acid, BCA; enhanced chemiluminescence, ECL; macrophage colony stimulating factor, MCSF

## Competing interests

The author(s) declare that they have no competing interests.

## Authors' contributions

JEM designed the study, participated in animal experimentation and tissue collection, participated in the protein isolation, suspension bead array assays, ELISA, statistical analysis and drafted the manuscript. LO performed the routine histopathology interpretations and imaging. JC participated in protein isolation, suspension bead array assays and created the associated data charts. RS peer-reviewed the data interpretations. All authors read and approved the final manuscript.
